# Enhancement of the photoelectric performance of dye-sensitized solar cells using Ag-doped TiO_2 _nanofibers in a TiO_2 _film as electrode

**DOI:** 10.1186/1556-276X-7-97

**Published:** 2012-02-02

**Authors:** En Mei Jin, Xing Guan Zhao, Ju-Young Park, Hal-Bon Gu

**Affiliations:** 1 Department of Electrical Engineering, Chonnam National University, Gwangju, 500-757, South Korea; 2 Southwestern Research Institute of Green Energy Technology, Mokpo-si, Jeollanam-do, 530-400, South Korea

**Keywords:** dye-sensitized solar cell, TiO_2_, nanofiber, doping, solar conversion efficiency

## Abstract

For high solar conversion efficiency of dye-sensitized solar cells [DSSCs], TiO_2 _nanofiber [TN] and Ag-doped TiO_2 _nanofiber [ATN] have been extended to be included in TiO_2 _films to increase the amount of dye loading for a higher short-circuit current. The ATN was used on affected DSSCs to increase the open circuit voltage. This process had enhanced the exit in dye molecules which were rapidly split into electrons, and the DSSCs with ATN stop the recombination of the electronic process. The conversion efficiency of TiO_2 _photoelectrode-based DSSCs was 4.74%; it was increased to 6.13% after adding 5 wt.% ATN into TiO_2 _films. The electron lifetime of DSSCs with ATN increased from 0.29 to 0.34 s and that electron recombination was reduced.

## Introduction

Since the Grätzel group discovered dye-sensitized solar cells [DSSCs], many people became interested. The low-cost, high-solar conversion efficiency of DSSCs is considered as a possible alternative to the present silicon solar cells [1-3]. DSSCs employ a sensitizer (dye) adsorbed on a surface of a wide energy bandgap semiconductor and electrolyte dissolving redox couples such as I^-^/I_3_^- ^and platinum [Pt] counter electrode [4]. In DSSCs, the photoexcited electrons of the dye adsorbing on the TiO_2 _surface are transferred to the conduction band of TiO_2_, which are then taken to an outer circuit using a fluorine-doped tin oxide [FTO] substrate and a counter electrode, respectively, and then the electrons are passed to an electrolyte [5,6]. So, in order to get a high solar conversion efficiency in DSSCs, a high surface area for the porous TiO_2 _films for efficient absorption of the sensitizer and good networking between the particle to particle or particle to FTO substrate are very important [7-10]. So far, the TiO_2_-based DSSCs fabricated using multilayer approaches have shown the solar conversion efficiency of 11.3%, which is lower than the theoretical maximum (33%) [11,12]. So many research, in order to increase the solar conversion efficiency in DSSCs, have been studied about photoelectrodes such as synthesis of the wide bandgap of TiO_2_, the small particle size of 10 to approximately 20 nm, the wide surface area of TiO_2_, and the porosity. As stated above, these can increase the adsorption of dye, and by extension, the solar conversion efficiency could be increased [13,14].

In this study, DSSCs fabricated with a TiO_2 _nanofiber [TN] and an Ag-doped TiO_2 _nanofiber [ATN] were used to increase the TiO_2 _film's surface area for dye adsorption. The study has discussed the electrochemical properties of the TN-added cells or the ATN-added cells by photocurrent-voltage curves.

## Experiment

### Preparation of TN and ATN

TN was fabricated using the electrospinning technique [15]. The electrospinning technique has been recognized as a versatile and effective method for the production of fibers with small diameters and with high surface-to-volume ratio [16-18]. It is demonstrated that titanium isopropoxide [TiP] can be added directly to an alcohol solution containing polyvinylpyrrolidone [PVP] (with a molecular weight [MW] of 1,300,000). To suppress the hydrolysis reaction of the sol-gel precursor, acetic acid as well as PVP solution in ethanol must be added. TiP of 6 mL was mixed with 12 mL acetic acid and 12 mL ethanol. After 60 min, this solution was added to 30 g ethanol that contained 10 wt.% PVP and 1.986 mL of 0.5-N AgNO_3 _(5% TiP mol), followed by magnetic stirring for 24 h. The spinning solution underwent electrospinning with an applied voltage of 20 kV, a flow rate of 50 μL/min, and a tip to collector distance of 15 cm. The prepared electrospun fiber was calcinated at 500°C.

### Preparation of the TiO_2 _photoelectrode and the Pt electrode

TiO_2 _paste was prepared by mixing nitric acid-treated and nanosized TiO_2 _(P-25, Degussa, Evonik Industries, Essen, Germany) powder with acetyl acetone, nitric acid, ethanol, distilled water, Triton X-100, and polyethylene glycol (Junsei Chemical Co., Ltd., Chuo-ku, Tokyo, Japan; average MW 20,000) binders for 10 h at 300 rpm by using the Planetary Mono Mill (pulverisette 6, Fritsch GmbH, Idar-Oberstein, Germany). In this process, the TiO_2 _powder was treated with nitric acid. The 12-g TiO_2 _(P-25) powder was mixed with distilled water and nitric acid (*v*/*v*, 120:1) at 80°C for 8 h using a hot plate. After mixing, the TiO_2 _nitric acid solution was dried at 100°C for 24 h. The prepared TiO_2 _paste was cast on pre-cleaned FTO (Pilkington FTO glass, Nippon Sheet Glass Co., Ltd., Minato-ku, Tokyo, Japan; 8 Ω/cm^2^) using the squeeze printing method. The coated TiO_2 _films were sintered at 450°C for 30 min. The active area of the TiO_2 _film was 0.25 cm^2^. The TiO_2 _film was immersed into a 5 × 10^-4^-mol/L ethanol solution of Ru(dcbpy)_2_(NCS)_2 _(535-bis, Solaronix Co., Aubonne, Switzerland) overnight, then rinsed with anhydrous ethanol, and finally dried. The counter electrode was prepared using the squeeze printing technique and subsequently sintered at 450°C for 30 min. The counter electrode material was a Pt catalyst (Solaronix Co.).

### Assembly of the testing cells

The Pt electrode was placed over the dye-adsorbed TiO_2 _electrode, and the edges of the cell were sealed. The sealing was accomplished by hot-pressing two electrodes together at 120°C. The redox electrolyte was injected into the cell through two small holes drilled in the counter electrode. The redox electrolyte was composed of 0.3 mol/L 1,2-dimethyl-3-propylimidazolium iodide (Sigma-Aldrich Corporation, St. Louis, MO, USA), 0.5 mol/L 4-tert-butylpyridine (Sigma-Aldrich Corporation), and 3-metoxypropionitrile as solvent. The holes were then covered and sealed with a small square of sealing material and microscope objective glass.

### Measurements

The crystalline phase of the prepared TN and ATN was obtained by high resolution X-ray diffractometry [XRD] (D/MAX Ultima III, Rigaku Corporation, Tokyo, Japan) using CuKα radiation, and field-emission scanning electron microscopy [FE-SEM] (S-4700, Hitachi High-Tech, Minato-ku, Tokyo, Japan) and energy dispersive X-ray spectrometry [EDX] (EMAX Energy EX-200, HORIBA Ltd., Minami-Ku, Kyoto, Japan) were used to examine the morphology and chemical element analysis of the TiO_2 _film.

In order to investigate the physical and optical characteristics of the natural dyes, the UV-visible spectrum measurement was performed. The photovoltaic properties were investigated by measuring the photocurrent-voltage characteristics under illumination with an air mass [AM] of 1.5 (100 mW/cm^2^) simulated sunlight. The charge transport characteristics were investigated by intensity-modulated photovoltage spectroscopy [IMVS]. The IMVS was measured using red light-emitting diodes [LED] (635 nm). The light intensities were modulated by 10% in a frequency range typically from 0.01 to 100 Hz.

## Results and discussion

**Figure 1:**
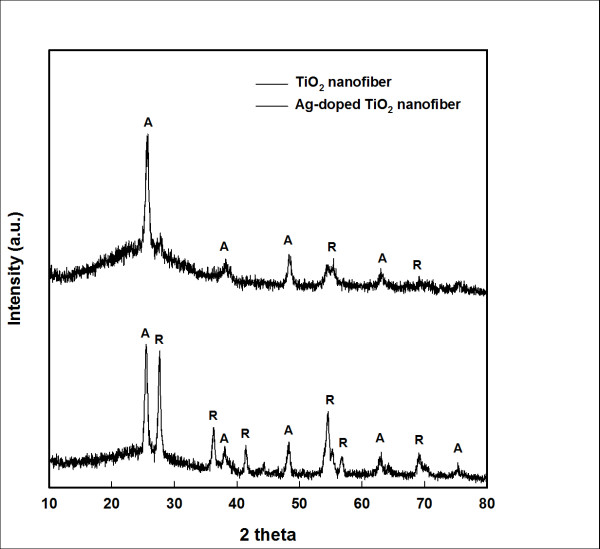
**XRD patterns of ATN and TN**.

Figure 1 shows the XRD patterns of pure TN and ATN at a calcination temperature of 500°C. In a study by Park et al. [19], silver had a down phase transition temperature to that of TN. The anatase ratio of the corresponding plane (101) extracted from the XRD pattern was calculated using the Spurr equation against the corresponding plane (101) [20]. Pure TN was only observed on the anatase phase; ATN was observed on both the anatase (46%) and rutile (54%) phases.

**Figure 2:**
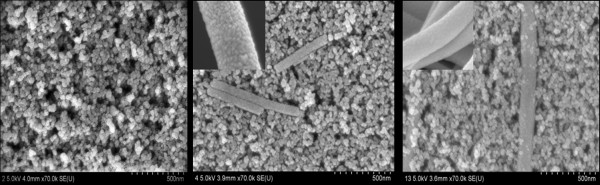
**FE-SEM images**. Pure TiO_2 _film and TiO_2 _films with TN and ATN.

The surface morphologies of the pure TiO_2 _photoelectrode and TiO_2 _photoelectrode with TN or ATN TiO_2 _films were obtained by FE-SEM and are depicted in Figure 2. The pure TiO_2 _film observations show very good film surface uniformity with about 25 nm TiO_2 _nanoparticles and thin film porosity. TN and ATN nanofibers can be observed at the surface of the film, so the nanofiber-added TiO_2 _film has an advantage to having higher adsorption of dye molecules and also supports the penetration of the I^-^/I_3_^- ^redox couple into the TiO_2 _film. Moreover, the surface area of the TiO_2 _films was larger, so the dye molecule adsorption space was also larger. Consequently, the increased surface absorption enhanced the solar energy conversion efficiency.

**Figure 3:**
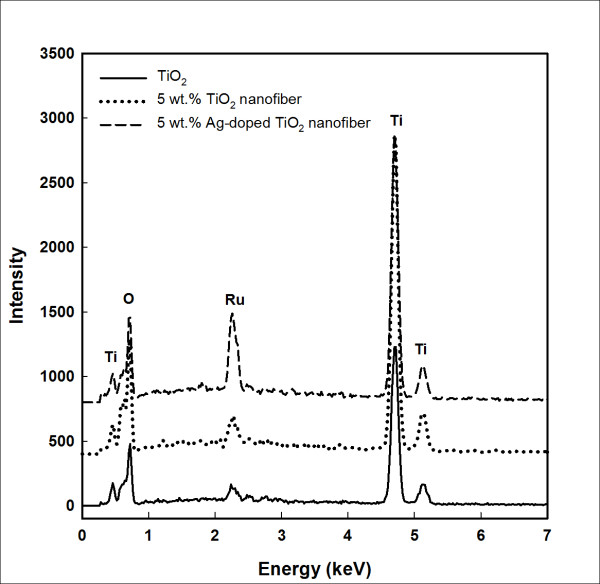
**EDX of TiO_2 _films**. Pure TiO_2 _film (straight line) and TiO_2 _films with 5 wt.% TN (dotted line) or 5 wt.% ATN (broken line).

Figure 3 and Table 1 show the EDX results of pure TiO_2 _films with 5 wt.% TN or ATN. It was found that the distribution of TN and ATN on pure TiO_2 _films and on the Ru element has increased. Figure 3 shows that the Ru peak energy around 2.2 keV was higher in the 5 wt.% ATN than those of other samples and the weight of Ru is 0.42 at.%. So, the ATN on the TiO_2 _film gave a higher adsorption of dye (Ru) molecules and also supported electron transfer in the TiO_2 _film. Consequently, increased adsorption of dye and electron transfer enhanced the solar energy conversion efficiency.

**Table 1:** EDX data of pure TiO_2 _films and TiO_2 _films with 5 wt

Compound	Pure TiO2 (total = 100)		5 wt.% TN (total = 100)		5 wt.% ATN (total = 100)	
	wt.%	at.%	wt.%	at.%	wt.%	at.%
OK	46.77	72.62	47.03	72.89	43.24	70.00
TiK	52.39	27.17	51.81	26.82	54.39	29.41
RuL	0.84	0.21	1.17	0.29	1.62	0.42
AgL	-	-	-	-	0.75	0.18

**Figure 4:**
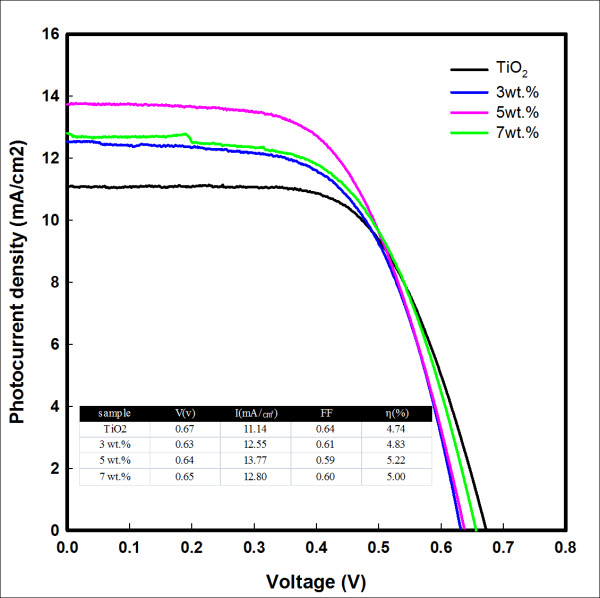
**Photocurrent-voltage characteristics of TiO_2 _film with different amounts of TN**.

Figure 4 shows the photocurrent-voltage characteristics of a sandwich solar cell based on a TiO_2 _film with different amounts of TN. The solar cell irradiated with a 1,000-W xenon lamp with a light intensity of 100 m/cm^2 ^as a light source. The short-circuit current density [*J*_sc_] and the open circuit voltage [*V*_oc_] values of the solar cell on pure TiO_2 _film are 11.14 mA/cm^2 ^and 0.67 V, respectively. The fill factor [FF] value is 64%, and the solar energy conversion efficiency [*η*] value is 4.74%. The *η *of the TiO_2 _film with 5 wt.% TN is higher than those with other contents (such as 3 wt.% and 7 wt.%), and *V*_oc_, *J*_sc_, FF, and *η *values are 0.64%, 13.77 mA/cm^2^, 59%, and 5.22%, respectively.

**Figure 5:**
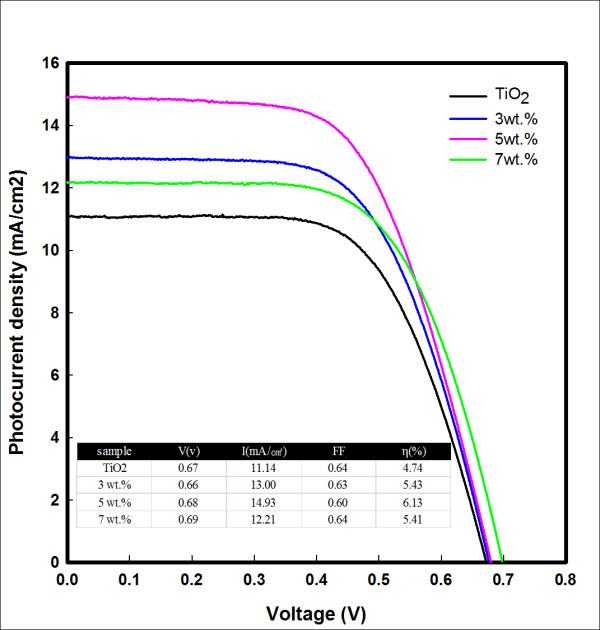
**Photocurrent-voltage characteristics of TiO_2 _film with different amounts of ATN**.

Figure 5 shows the photocurrent-voltage characteristics of DSSCs sensitized with different amounts of ATN. The *η *of the TiO_2 _film with 5 wt.% ATN was the best at 6.13%; the ATN on a nanocrystalline TiO_2 _film enhanced the charge recombination, and there was a 129% improvement in the photovoltaic device solar conversion efficiency.

**Figure 6:**
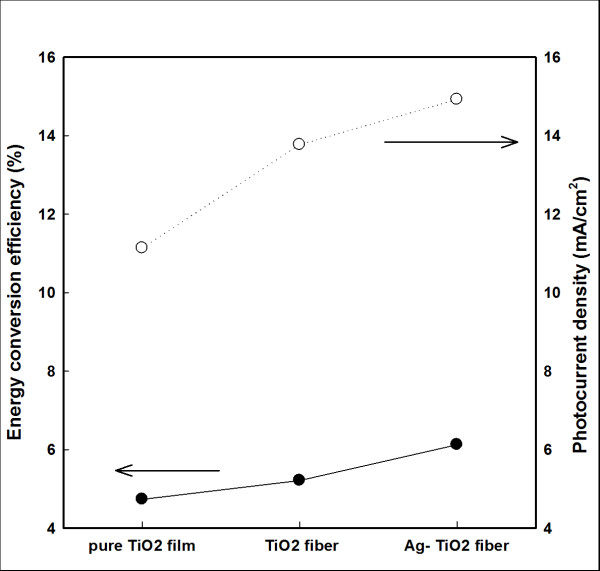
**Photocurrent density and *η *of pure TiO_2 _films and with 5 wt.% TN and ATN**.

Figure 6 shows the photocurrent density and *η *of pure TiO_2 _films, TiO_2 _films with 5 wt.% TN, and TiO_2 _films with 5 wt.% ATN. The photocurrent density and *η *of DSSCs with 5 wt.% ATN were the best among the samples.

**Figure 7:**
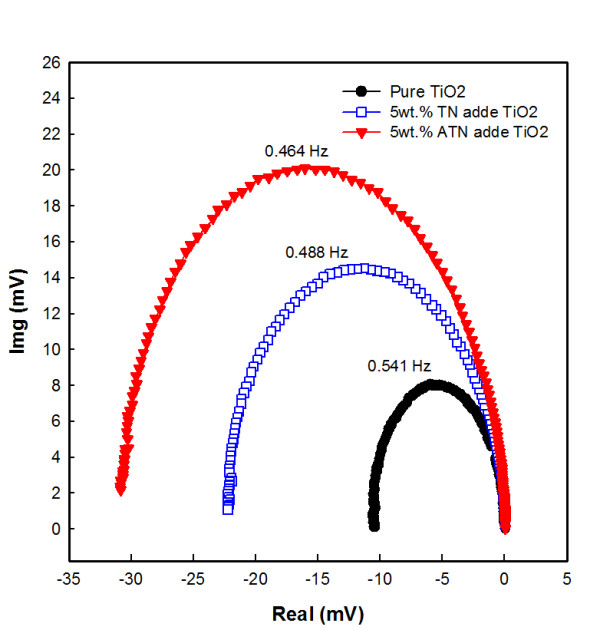
**DSSCs' intensity-modulated photovoltage spectroscopy results**. Pure TiO_2 _film (black filled circle) and TiO_2 _films with 5 wt.% TN (blue empty square) or ATN (red inverted filled triangle).

The electron lifetimes within DSSCs are determined primarily by the recombination of electrons with iodine, electrolyte, and oxidized sensitizers. To understand the recombination lifetime, *T*_n _was determined using the relation $T_{\text{n}}\text{ = }\frac{1}{2\pi f_{\text{min}}}$, where *f*_min _is the frequency giving the lowest imaginary component in IMVS. So, we are using IMVS to analyze the lifetime of DSSCs with ATN; the charge transport characteristics were investigated by IMVS. The IMVS are shown in Figure 7. IMVS was measured using LED (635 nm). The light intensities were modulated by 10% in a frequency range typically from 0.01 to 100 Hz. The electron lifetime was increased by adding ATN, and this sample had the highest photovoltage compared with the others. The results are consistent with the photocurrent-voltage curves. The electron lifetime of DSSCs with ATN increased from 0.29 to 0.34 s. This result clearly indicates that electron recombination with the oxidized species is reduced by adding ATN in the TiO_2 _film. This can be understood by either looking at the improved connection of TiO_2 _nanoparticles or the Ag effect of the electrons during transition. The increased electron lifetime and the reduction of the electron transit time can explain the increment of *J*_sc _by the addition of ATN.

## Conclusions

In conclusion, TN and ATN were added into the TiO_2 _film of DSSCs. An enhanced *η *of 129% was achieved from the 5 wt.% ATN concentration. The added ATN had also contributed toward the enhancement of dye adsorption as seen from EDX results, and surface area was increased by the fibers. It gives many absorption sites for the dye, and the ATN that was added to the TiO_2 _film enhanced the charge recombination. The study has shown that the performance of DSSCs can be strongly improved using fibers. An *η *of approximately 6.13% has been achieved for DSSCs with ATN at the irradiation condition of AM 1.5 (100 mW/cm^2^) simulated sunlight, and *J*_sc_, *V*_oc_, and FF are 14.93 mA/cm^2^, 0.68 V, and 60%, respectively. It is understood that the lifetime of DSSCs was increased by the addition of the ATN and that electron recombination was reduced.

## Competing interests

The authors declare that they have no competing interests.

## Authors' contributions

EMJ participated in the fabrication of DSSCs and in the analysis of photocurrent-voltage characteristics. XGZ was involved in the FE-SEM, EDX, and IMVS analyses of TiO2 films and TN- and ATN-doped TiO2 films. J-YP participated in the preparation of TN and ATN, and in the analyses of XRD and FE-SEM results. H-BG is the thesis director. All authors read and approved the final manuscript.
